# Is it possible to lower the incidence of central venous catheter infections in poor countries?

**DOI:** 10.1186/2197-425X-3-S1-A886

**Published:** 2015-10-01

**Authors:** A Jukic, G Simic, V Nikolic, S Petrovic

**Affiliations:** Anesthesiology and Intensive Care, The National Cancer Research Center, Belgrade, Serbia; Medical Oncology, The National Cancer Research Center, Belgrade, Serbia

## Introduction

Central venous catheter (CVC) infections are a major cause of nosocomial infections and patient mortality. Although much has been done in recent years in order to lower the incidence of CVC infections, not all the means are available in poor countries. This research shows that it is possible to get closer to achiving this goal by applying recommendations which do not require significant financial expenditures.

## Objectives

The purpose of this retrospective cohort study was to establish whether it is possible to lower the incidence of CVC infections by using recommendations of the Infectious Diseases Society of America (IDSA) and World Health Organisation Prevention of Hospital Infections by Intervention and Training (PROHIBIT) programme which do not require significant financial means. These included education and training of the anesthesiologists and nurses in how to mantain aseptic conditions for insertion and usage of CVC.

## Methods

We concentrated on applying recommendations and changing common errors in daily routine of preparation for insertion, insertion, usage and removal of CVCs. We did not use new catheters with an anti-infective surface, chlorhexidine antiseptic solutions, topical anti-infective creams or transparent adhesive dressings which are either still not available in our country or are more expensive than previously used measures.Educational and training programmes were started from the beginning of 2013. The study included 164 patients who had a standard triple-lumen polyurethane catheters inserted into the internal jugular vein at the National Cancer Research Center of Serbia from January 2010-December 2012 and January 2013-February 2015. The data were analysed using a X² statistical test.

## Results

Catheters were removed after an average of 14 days (3-28). The primary outcome measurement for this study was the culture of the catheter tip determined by the semiquantitive method after the removal of catheter. The incidence of positive catheter-tip cultures placed from 2010-2012 was 52% while for the period 2013-2015 it was 35%. There was a statistically significant reduction of positive cultures in catheters placed after 2013. when we started applying these measures. (X^2^ = 4.58, DF = 1, p = 0.032).

## Conclusions

Study results show that it is possible to lower the incidence of CVC infections by education and training of anesthesiologists and nurses who take part in insertion, maintenance and usage of CVCs.
